# Proteomic Approaches and Potential Applications in Autosomal Dominant Polycystic Kidney Disease and Fabry Disease

**DOI:** 10.3390/diagnostics13061152

**Published:** 2023-03-17

**Authors:** Merita Rroji, Andreja Figurek, Goce Spasovski

**Affiliations:** 1 Department of Nephrology, Faculty of Medicine, University of Medicine Tirana, 1001 Tirana, Albania; 2 Institute of Anatomy, University of Zurich, 8057 Zurich, Switzerland; 3 University Clinic for Nephrology, Medical Faculty, University St. Cyril and Methodius, 1000 Skopje, North Macedonia

**Keywords:** chronic kidney disease biomarker, proteomic, extracellular vesicles

## Abstract

Although rare, hereditary diseases, such as autosomal dominant polycystic kidney disease (ADPKD) and Fabry disease (FD) may significantly progress towards severe nephropathy. It is crucial to characterize it accurately, predict the course of the illness and estimate treatment effectiveness. A huge effort has been undertaken to find reliable biomarkers that might be useful for an early prevention of the disease progression and/or any invasive diagnostic procedures. The study of proteomics, or the small peptide composition of a sample, is a field of study under continuous development. Over the past years, several strategies have been created to study and define the proteome of samples from widely varying origins. However, urinary proteomics has become essential for discovering novel biomarkers in kidney disease. Here, the extracellular vesicles in human urine that contain cell-specific marker proteins from every segment of the nephron, offer a source of potentially valuable urinary biomarkers, and may play an essential role in kidney development and kidney disease. This review summarizes the relevant literature investigating the proteomic approaches and potential applications in the regular studies of ADPKD and FD.

## 1. Introduction

Chronic kidney disease (CKD) is highly prevalent and has become one of the world’s major non-communicable causes of mortality. It is foreseen that it will eventually affect more people and become more significant among all other worldwide causes of mortality. Additionally, CKD lowers the quality of life and has a profound socioeconomic impact [1].

Despite the rarity of any inherited kidney disease (IKD), recent research suggests that 20% of children and 10% of adults with CKD have genetic variations that can be identified as disease-causing [2]. Autosomal dominant polycystic kidney disease (ADPKD) is the only hereditary kidney disease represented in national and international registries. While the incidence of ADPKD is similar worldwide, its relative frequency among CKD causes varies depending on the prevalence of other lifestyle-related nephropathies, such as diabetic and hypertensive kidney disease. The additional IKDs are categorized as unknown in the ERA-EDTA registry’s “miscellaneous” category and the USRDS. As a result, there is an issue with invisibility: out of sight, out of mind [3]. CKD is a complex, heterogeneous disease influenced by both genomic and environmental factors, with a possibility of ruling out IKD by genetic testing. The familial aggregation of CKD can be explained by genetics in two main ways: Mendelian gene pathogenic variations are rare but significantly impact disease development; examples include ADPKD [4].

Moreover, common genetic variations are quite prevalent and only have a minimal impact on the phenotype. In addition, to the advancement of genomic medicine, informed consent, the potential advantages, and implications of genetic results, and the present restrictions on the interpretation of genetic results may identify the disease but not always portray the progress of the disease [5].

In clinical settings where estimate GFR (eGFR) equations are routinely used, the key limitations remain to be the limited sensitivity in detecting early CKD and poor prediction of the CKD development. Recent research by Rodrigues et al. revealed that the mistakes of eGFR formulas in patients with ADPKD were huge, frequent, and unexpected. The average inaccuracy of each calculation was around 50 percent of actual renal function. This extensive variation was observed for each evaluated equation based on creatinine or cystatin-c. Due to the high variability of eGFR, patients may be misplaced in higher or lower stages of CKD. This makes it hard to obtain an accurate picture of renal dysfunction, CKD stage, and how renal function changes over time and find people who might benefit from treatment. In this regard, whenever feasible a measured GFR is recommended for ADPKD patients [6]. In addition to eGFR, established clinical biomarkers, such as albuminuria, serum creatinine (sCr) levels, or the urine albumin-to-creatinine ratio (UACR) can detect CKD. However, their ability to predict individual CKD risks or the likelihood of developing the end-stage renal disease (ESRD) is rather limited. 

Hence, there is a demanding need to develop widely accessible methods, to have a specific, reproducible, longitudinally applicable, and predictable tool for monitoring and predicting individual progression rates and, therefore, the likely initiation of renal replacement therapy (RRT) in patients with inherited disease [7]. This necessity has recently been reflected in clinical research, emphasizing the identification and characterization of multiple biomarkers (proteins, peptides, and miRNA) in different bodily fluids using high-resolution technologies for future biomarker-guided therapies [8,9]. Urinary biomarker assays meet these needs with the advantage of being non-invasive and convenient [10]. One method that may address fundamental research and practical medicinal issues is mass spectrometry (MS)-based proteomics [11]. Over the last several decades, proteomic approaches and cutting-edge laboratory equipment have been demonstrated to play a significant role in exposing essential molecular insights about the disease processes [12]. Nowadays, the term “proteomics” is increasingly used to describe complex and extensive analyses of proteins [13]. Knowing that proteome profiles, in addition to the clinical and histological evaluation, may potentially identify altered molecular pathways in CKD, it is clear that their integration into standard clinical practice is an unmet need. Apart from its use to diagnose diseases, proteomics can be used to track their courses. This review summarizes the urine proteomic research and discusses its application to two significant IKD disorders, i.e., ADPKD and Fabry disease. Recent advancements in human medicine may facilitate the resolution of outstanding biology and medical concerns about IKD and open the avenue for the development of innovative and causative therapeutics.

### Proteomics

Currently, 12,252 proteins compose the proteome of healthy adult human kidney tissue and it is feasible to characterize the whole human proteome due to the sensitivity and acquisition speed of contemporary mass spectrometers. The Human Protein Atlas project, which attempts to map human tissue proteomes using genome-wide antibody-based profiling on tissue microarrays, has identified 13,345 protein-coding genes in the kidney [14]. According to known kidney functions, both investigations demonstrate that the majority of elevated genes and proteins in the kidney are involved in the transportation of small molecules [15].

From the perspective of renal pathology, the deoxyribonucleic acid (DNA) sequence may be mainly seen as static and homogeneous across the body, even though somatic mutations obviously occur. The proteome, however, is quite dynamic; in fact, this is one of the key reasons why studying these domains is important for understanding the “functional” effects of the genome. Additionally, because the proteome varies across the body, different findings will be obtained from proteomic analyses of the liver, muscle, kidney, blood, urine, etc. Additionally, heterogeneity exists within tissues. For instance, the kidney’s glomerular, endothelial, and tubular cells are heterogeneous [16]. Examining the proteome has therefore obvious benefits since, in contrast to the genome, it offers information about biology at a certain moment and location that is “proximal” to the illness manifestation. Proteomic biomarkers are defined as a particular peptide or protein that is linked with a specific condition, such as the beginning, presentation, or the course of a disease, or the efficacy of the therapy. Blood and urine are accessible biofluids that are vital for current diagnostic and prognostic techniques in clinical nephrology. Perhaps most significantly however, is the fact that proteins are modifiable and can thus be targeted for treatment follow-up [17,18].

Urine is one of the most useful sample sources for identifying illness biomarkers. When compared to other biological fluids, urine collection is straightforward, non-invasive, and has a comparatively large amount of accessible fluid. One of the most challenging items about utilizing urinary proteomics is that the urinary proteome is made up of proteins and peptides from plasma, secreted proteins, microvesicles, and whole cells from the entire genitourinary tract [9,19]. Different protein profiles and abundances are routinely seen for a variety of diseases. Furthermore, there is a lot of variation in the urine proteome based on factors, such as age, sex, diet, and physiological state [20]. Therefore, although used in most of the research on urinary proteome to evaluate kidney function, it has never been thoroughly established whether the amounts of proteins in the urine correlate with kidney tissue levels [9].

Extracellular vesicles (EVs) are membrane-bound nanostructures that are released into extracellular fluid when cells are stressed. Intracellular communication is one of their most essential tasks, in which they send cargo to target cells, changing their phenotype [21,22]. Some of them are proteins, lipids, DNA, messenger RNA (mRNA), and microRNA (miRNA), and the majority have been studied as biomarkers [23]. Surface signs of EVs include aquaporin-2 (AQP2) from the collecting duct, sodium/hydrogen exchanger-1 from the proximal tubule, and podocalyxin from the podocytes. Exosomes, microparticles, and apoptotic bodies are the three primary EV types. Their cellular origin, size, and payload may all be used for their identification [24]. EVs have been discovered in several biological fluids, including urine and blood. Even though circulating EVs do not seem to be able to cross the filtration barrier, proteomic analysis has shown that most of the cells in the cargo of urinary EVs are glomerular, tubular, prostate, and bladder cells. This supports the idea that urinary EVs mainly come from cells in the genitourinary tract that face the urinary space. Because changes in the quantity or nature of released EVs may be connected to the onset of the disease or the effectiveness of treatment, assessment of urine EVs may be a rational and unique diagnostic strategy in renal disease [25,26].

## 2. ADPKD

With a high penetrant state, affecting between 1:400 and 1:1000 live births, ADPKD is the most common inherited kidney disease. The bilateral kidney cysts that characterize ADPKD continuously grow and develop, inducing progressive renal enlargement and increasing the total kidney volume (TKV), frequently complicated by hypertension, polyuria, nocturia, discomfort, nephrolithiasis, hematuria, infections, kidney function loss, and ESRD which typically manifests at age 55 [27,28]. Even within families, disease severity and course might vary considerably. The two most frequent loci are PKD1 located on chromosome 16p13.3 (78% of cases), and PKD2 located on chromosome 4q21 (15% of cases), whereas minor loci are responsible for a small proportion of frequently abnormal patients and a minority of cases with unresolved genetic defects [29,30]. The course of the disease is difficult to predict due to the unique compensating capability of polycystic kidneys, which is maintained through hyperfiltration of residual nephrons where kidney function is preserved despite the significant cystic expansion [28,31]. Late in the course of the disease, a noticeable rise in serum creatinine is often followed by a steady, rapid drop in GFR and progression to ESRD, at which point it is usually too late to try to treat the disease [32]. Current methods of diagnosis, including ultrasonography and magnetic resonance imaging (MRI), are widely accepted in guidelines together with the family history and genetic analysis for diagnosis of the disease. However, these investigations do not always adequately address the common challenges, complications, and correlate well with disease progression or response to therapy in ADPKD. In addition, height-adjusted total kidney volume (htTKV) emerged as a potentially relevant biomarker with decreased renal function [33,34].

Due to the substantial advancement in our understanding of cyst development and expansion biology, the ability to detect, assess, and predict disease severity in patients with ADPKD has significantly improved as an attempt to identify non-invasive markers of the disease. Hence, more than a thousand proteins have been found in the current analysis of the human urine, and furthermore studying EV proteome, has gained significant research attention for establishing disease-related biomarkers due to their involvement in the pathophysiology of the disease and renal function.

Following attempts for identifying and validation of urine biomarkers in different kidney diseases, Kirstler and his group, [35] firstly identified a urinary proteomic ‘footprint’ of ADPKD. Their research shows that ADPKD patients have a unique profile of proteins in their urine that makes them different from healthy controls and people with other kidney diseases or problems with the urinary system. Out of 197 proteins with significantly altered urine excretion, they described 38 with amino acid sequences identified, the majority of which were fragments of collagen type I or III.

It was revealed that extracellular matrix (ECM) adaptive modifications are essential for cyst growth in ADPKD, and polycystic kidney disease-related investigations have reported anomalies in the ECM [36]. The matrix metalloproteinase inhibitor batimastat has been shown to diminish cyst formation in (cy/+) rats, a rodent model of polycystic kidney disease, and serum levels of these enzymes have been notably higher in ADPKD patients [37] Moreover, they identified a shared mechanism in ADPKD and renal aging [38]. In addition, uromodulin peptides, which have been linked to the tubular damage in the past, were also found in the urine samples [39].

The same author reported a clinical proteomic study analyzing the samples from the CRIPS study urine samples showing that young individuals with ADPKD who have mutations, but no visible cysts may be identified using urine proteome analysis, underlining that further attempts are needed to refine the model. The authors hypothesized that cyst formation induces the organization of the renal ECM, which in turn slows down the normal collagen breakdown and, ultimately, causes a significant drop in collagen-derived peptides in ADPKD patients’ urine and the decline is inversely related to each person’s height-adjusted total kidney capacity. This may suggest collagen fragments as a prognostic indicator [40]. Fibrinogen alpha chain and keratin peptides were found to be more present in ADPKD samples, which is consistent with the fact that fibronectin and keratin 19 speed up renal cystogenesis and are linked to ADPKD [41].

They also noticed a consistent downregulation of uromodulin’s c-terminal segments associated with ADPKD, possibly due to less efficient uromodulin breakdown. Additionally, osteopontin levels were higher [42] due to the decreased excretion of an osteopontin fragment in urine. Testing the diagnostic biomarker model in a cohort of 481 individuals with a range of renal and extrarenal disorders, including acute kidney injury (AKI), they reported that it was particular to ADPKD. The markers previously linked to AKI were among the proteome changes in ADPKD [43,44,45,46].

Later, the same group reported that a cohort of patients between the ages of 24 and 46 might be predicted to develop ESRD within 10 to 13 years of follow-up using a biomarker-based classifier of 20 urine peptides at baseline [47]. The effectiveness of the biomarker score was equivalent to that of the htTKV, and the prediction accuracy for the low or high risk progression to ESRD was higher when the two variables were combined compared to their separate use. The same biomarker model predicted an 8-year reduction in GFR of 30 mL/min/1.73 m^2^ in young individuals (24 years at baseline). They were able to identify eighty percent of the prognostic peptides showing that these are the products of large proteins’ endogenous proteolytic cleavage. Therefore, in addition to being reliant on the parental proteins’ expression, the activity of the proteases that produce them also plays a significant role in their excretion in urine. Nine proteases were discovered as a result of in silico investigations of putative proteolytic pathways entangled in the production of the predicting peptides, including cathepsins D, E, and L, meprin A, matrix metallopeptidase 2 (MMP2), 3, 8, and 9, and pepsin A. Due to their involvement in the ECM turnover that occurs during cyst growth, MMPs may play a role in ADPKD. They observed a shift in the activity of the cathepsins from cathepsin L, whose activity was decreased, to cathepsin D and E. (which showed increased activity). In mouse Pkd, null kidneys and human ADPKD cells decreased cathepsin L activity has been observed and may contribute to cyst formation by reducing the proteolytic processing of Cux1, a homeobox gene that suppresses the cyclin kinase inhibitors p21 and p27 [48]. 

Recently, Rauniyar N et al. identified 69 urinary target proteins that were found significantly dysregulated in ADPKD and could be used to classify ADPKD patients into groups more or less similar to normal controls, proposing that this approach could be further longitudinally validated and used to provide urinary protein biomarkers of the cyst growth rate in ADPKD. However, further validation studies in larger patient cohorts are crucial to refine the biomarker panel and establish its relevance in ADPKD progression [49].

Furthermore, clinical nephrology research on urinary EVs (uEVs) is developing quickly. The urine EV proteome may now be subjected to quantitative and qualitative investigations, which is essential for comparing the protein expression patterns of samples derived from various sources. Comparing uEVs collected from ADPKD patients to uEVs isolated from healthy controls, many proteins have their expression altered [50]. Analyzing a urine test that assesses the urine exosomal polycystin-1(PC1)/ transmembrane protein 2 (TMEM2) or polycystin-2 (PC2)/TMEM2 ratio may be possible to diagnose and track polycystic kidney disease [51]. A defective copy of either PKD1 or PKD2, which encodes polycystin-1 (PC1) or polycystin-2 (PC2), is inherited by individuals with ADPKD, in which PC1 and PC2 are released in urinary exosome-like vesicles (uELVs), and PC1 is present in a fragmented form and may bind with PC2. PC1 and PC2 levels were lower in PKD1 mutation carriers compared to healthy individuals. Additionally, in those with PKD1 mutations, TMEM2, a protein with fibrocystin homology, was found to be 2.1-fold greater. Although it has been demonstrated that people with PKD1 mutations have lower PC1 or PC2 to TMEM2 ratios, the mechanism is presently unknown. The PC1/TMEM2 ratio in the research cohort was found to have an inverse relationship with the htTKV. People who have poor outcomes may have low PC1/TMEM2 ratios from the start. This may be influenced by the type of mutations that an individual has in the PKD1 gene, which is responsible for producing the polycystin-1 (PC1) protein. Individuals with a null PKD1 allele, which is a mutation that completely prevents the production of functional PC1 protein, may have a more severe form of PKD. Here, PC1 is unable to load into uELVs. People with a good prognosis may have a missense or hypomorphic mutation, which lets them load a lot of PC1 into uELVs from the defective allele, which may help to mitigate the effects of the mutation.

Nevertheless, it has been demonstrated that EVs from ADPKD patients have considerably higher expression levels of proteins involved in the cytoskeletal structure (ezrin, radixin, moesin (ERM) family, and annexin 2), which connect the actin cytoskeleton to the plasma membrane. Cyst formation and AQP2 intracellular trafficking are both impacted significantly by cytoskeletal reorganization. The observed decrease in AQP2 levels in ADPKD patients is consistent with the patients’ decreased capacity for concentrating their urine [52]. Most importantly, it was additionally shown that there were considerable changes in the expression of PC1 and PC2, as well as other Ca2+-binding proteins (annexin A1, annexin A2, protein S100-A9, protein S100-A8, and retinoic acid-induced protein 3).

The findings of this investigation support the hypothesis that uEVs may play a role in renal physiopathology, providing a unique way to track changes in the expression of proteins in the apical membrane of the urinary tract, which is hard to do without being invasive [53].

In ADPKD patients, it might be biologically possible to find the presence of villin-1, plakins, and complement in the uEV [54]. Villin-1 is an actin-modifying protein that affects the cell shape, actin rearrangement, and cell motility, mainly expressed in the proximal tubules’ brush border in the kidney [55]. Polycystin-1 is thought to have a role in controlling cell adhesion, migration, and actin cytoskeleton organization [56]. Villin-1 may be increased due to polycystin-1 abnormalities that cause cell polarity issues and aberrant cell proliferation.

Additionally, desmosomal plaque (also known as plakins) comprises several transmembrane proteins from the cadherin family and stabilizes the epithelial sheet by forming an adhesive connection at the basolateral membrane [57]. Polycystin-1 is necessary to develop cell polarity and as an anchor to the adhesion complexes for signal transmission, may be connected to desmosomes helping to maintain the cell adhesion, protein sorting, and cell polarity that are all disturbed in the cystic epithelium. Desmosomal proteins in ADPKD are mis-polarized from the basolateral side to the apical domain because PC1 no longer colocalizes with desmosomes [58]. This may help to explain why patients with ADPKD have increased plakin abundance in their uEVs. 

Moreover, is not clear why a complement may be found separated in ultracentrifuged urine [54,59]. The majority of complement proteins are large (exceeding 70 kDa) and unlikely filtered. Similarly, more logical explanation for the findings could be that renal epithelial cells make and remove the complement locally to kill bacteria, that in turn helps protect against urinary tract infections. ADPKD patients may have more complement system subunits in their uEVs because their kidney cyst epithelial cells are growing faster. Villin-1 and the plakins, in contrast to complement proteins, seem inappropriate for ADPKD early monitoring. They may be assessed, for example, to track the effectiveness of treatment, in more advanced stages of ADPKD [54].

MS through a proteome profile was utilized to identify the protein content of microvesicles and exosomes to differentiate medullary sponge–related cystogenesis from ADPKD [8]. Patients with ADPKD had urine full of proteins involved in cell growth and matrix remodeling. This is likely because pathological tissue remodeling causes cysts to form and grow. Conversely, patients with medullary spongiform kidneys, have systemic biochemical imbalances that can be reflected in their urine. This could explain why these patients are more prone to complications, such as parenchymal calcification/renal stones and bone mineralization defects [8]. Thirty-four reported core proteins differentiate between microvesicles and exosomes in medullary spongiform kidney and ADPKD. CD133 is highly expressed in the exosomes of patients with ADPKD compared to patients with medullary spongiform kidney disease and healthy controls. In addition to the E1A-stimulated gene 1 (CREG1), factors interacting with insulin-like growth factor 2 (IGF2) receptors to regulate cell proliferation were found in the exomes of ADPKD [60]. Additionally, in ADPKD, there are proteins needed to remodel the matrix, such as inter-alpha-trypsin inhibitor heavy chain 5 (ITIH5), and control the secreted salt, including [guanylate cyclase activator 2B (GUCA2B) or myelin and lymphocyte (MAL)]. Furthermore, the FAT atypical cadherin-4 protein is abundant in ADPKD patients’ exosomes. Loss of this protein stops kidney cells from dividing in a planned way, tubules from growing longer, and the dilatation of renal tubules [61].

All of these findings are markers of the crucial mechanisms for cyst formation and expansion, tubular cell proliferation, extracellular matrix abnormalities, and transepithelial fluid secretion into the cyst lumen which characterizes ADPKD. The main studies in the proteomic analysis in ADPKD are presented in the Table 1.

**Figure 1:**
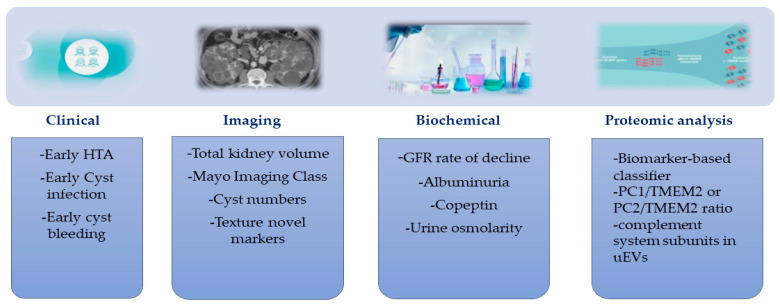
Potential biomarkers to evaluate disease severity in ADPKD urine exosomal polycystin-1(PC1)/ transmembrane protein 2 (TMEM2) or polycystin-2 (PC2)/TMEM2 ratio; urinary extracellular vesicles (EVs) [47,50,54].

One of the problems with treating ADPKD patients is the highly variable rate of disease progression. Even when the gene (PKD-1 or PKD-2) and the type of mutation that causes it are known, the path to ESRD is often unpredictable. A noninvasive biomarker to predict and to follow the lengthy treatment in ADPKD would have significant implications. The impact on progression and the magnitude of the effectiveness of the long-term tolvaptan therapy were assessed in ADPKD patients with urinary exosomal proteomics, identifying patients with the highest risk of rapid progression or poor treatment response [7]. A clear difference between rapid and slowly progressive profiles was seen in all stages of functional decline in ADPKD patients, where distinguished pathways and proteins included Notch, integrins, growth factor signaling, microtubule kinase, vesicle protein, and epidermal growth factor substrate. Comparative proteomic analysis of individual patient urinary exosomal proteins before and after four years of treatment with tolvaptan also identified different pathway modification patterns depending on the efficacy of the treatment response. Wnt signaling and upregulation of vesicle proteins were characteristic of urinary exosomes from ADPKD patients who responded well to tolvaptan. In contrast, urinary exosomes from ADPKD patients with poor responses showed upregulation of angiogenic signaling pathways and additional molecular forms of the vasopressin receptor AVPR2 [7,62]. Based on the previous research, the proteomic profiling of urinary exosomes would be considered with outstanding potential for developing a routinely and universally applicable method to identify and monitor ADPKD patients with rapidly progressive disease who are mainly at risk and who respond to tolvaptan therapy (Figure 1). Developing a urinary exosomal protein expression atlas will facilitate the identification of patients in need of treatment and most likely to benefit from long-term pharmacotherapy [19]. It is another step towards the goal of an increasingly individualized assessment of susceptibility to effective drug therapy and personalized medicine.

## 3. Fabry Disease 

The X-linked multisystem disorder Fabry disease (FD) is characterized by a lack of the lysosomal hydrolase -galactosidase A (α-GalA), which results in the progressive intralysosomal accumulation of globotriaosylceramide (Gb3) in various organs. The characteristic feature of FD is vasculopathy as a consequence of Gb3 deposition in vascular endothelial cells and subsequent inflammation and immune response. As a result, male FD patients experience life-threatening problems in their second to fifth decades of life, primarily chronic kidney disease, cerebrovascular events and hypertrophic cardiomyopathy [63]. The classical form of FD presents in early childhood with mild symptoms, whereas cardiovascular and ESRD occur in middle ages [64].

Current therapy of FD consists of enzyme replacement therapy (ERT), that reduces the accumulation of Gb3. However, FD patients’ plasma levels of these metabolites have shown vast variability, while the metabolite levels have weak correlations with the severity of the disease [65].

**Figure 2:**
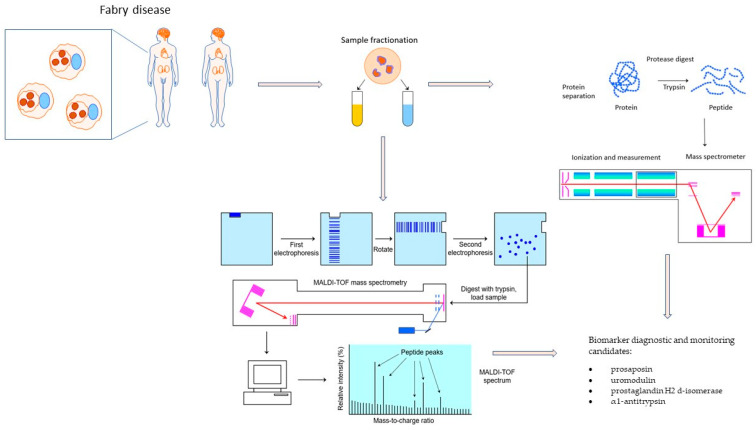
Proteomic approach in Fabry disease MALDI-TOF MS-matrix-assisted laser desorption/ionization time-of-flight mass spectrometry.

Measurement of the plasma α-GalA activity is used for diagnosis of the disease in male patients, but the measurement is not reliable in female patients since they have normal enzyme activity. The objectives have led to measurements of Gb3 concentrations in plasma and urine. Recent systematic analysis, however, has shown that Gb3 is not the best marker for determining the diagnosis or evaluating a patient’s response to treatment [66]. Gb3 may be normal in some patients and female heterozygotes when measured in random samples of whole urine [66]. Thus, another approach is needed to timely diagnose FD, particularly in female patients. Angiogenesis, fibrinolysis, oxidative inflammation, blood transport, and composition are all involved in the formation of FD pathology, and were previously studied to identify novel biomarkers in the plasma of FD patients [63]. Proteomic analysis is a novel method to learn how Fabry disease acts at the molecular level and finds potential biomarkers or therapeutic targets. One approach to proteomic analysis in Fabry disease is to use mass spectrometry (MS) to analyze protein expression levels and post-translational modifications (PTMs) in different tissues or biological fluids [Figure 2].

Peripheral blood mononuclear cells (PBMC) from FD patients and healthy individuals were used to identify multiple FD-specific biomarkers utilizing a proteomic analysis [67]. This study marked Rho GDP-dissociation inhibitor 1 and 2, calnexin, and chloride intracellular channel protein 1 as downregulated proteins in FD patients, whereas galectin-1, 14-3-3 protein zeta/delta, 14-3-3 protein theta, and g-enolase appeared to be upregulated in FD patients [67]. Protein folding, signaling, and cell growth are all impacted by calnexin and Rho GDP-dissociation inhibitor-1,2 [67]. While galectins are the regulators of an acute and chronic inflammation, g-enolase levels substantially rise in cardiovascular accidents and cerebral damage [67].

Heo et al. conducted a study in eight patients with classical FD where they analyzed plasma proteome profiles before and after ERT for a short-term (4–12 months) and a long-term (46–96 months) period [63]. Their research emphasized the complement pathway and interactions between β-actin (ACTB) and profilin-1 (PFN1) and endothelial nitric oxide synthase type 3 (eNOS or NOS-3), as possible indicators of the pathophysiology of FD. ACTB is a universal component of all eukaryotic cells and participates in a number of cellular functions, including motility, structure, and integrity. Recent research has also indicated that ACTB is crucial to the control of eNOS [63]. Actin-binding protein PFN1 controls actin polymerization in response to signals from the outside of the cell [68]. In the same study, the plasma levels of untreated FD patients had elevated levels of C3 and C4, and both levels gradually dropped with ERT, although C1qc level remained constant [63]. iC3b was the only protein, in contrast to the varied levels of C4b, that exhibited a gradual drop in level during the longer term of ERT. As a result, monitoring iC3b levels during an ERT course may help assess the therapeutic effectiveness of ERT in FD patients. Its gradual variations over the extended time of ERT were notable since they were akin to Gb3, which is often used as a plasma biomarker. Additionally, its alterations had a greater correlation with the total dose of ERT compared to plasma Gb3 [63].

In a small study conducted by Capasso et al., they used a proteomic approach to detect early markers of Fabry disease for diagnostic purposes and therapeutic monitoring. They discovered several proteins that can distinguish between the urinary proteome profiles of healthy participants and all naive patients affected by FD. Prosaposin, a protein precursor made up of four short peptides that act as sphingolipid hydrolase activators, is the potential indicator that has been found as very interesting one [66]. Furthermore, Ig kappa chain V-III and prostaglandin H2 d-isomerase are two examples of proteins that were discovered to be downregulated in this analysis [66]. It was also found that uromodulin was upregulated in the urine of Fabry patients, in contrast to what had been already described in the literature. Since the creatinine and GFR levels for all patients were nearly normal, the uromodulin upregulation may serve as an early indicator of tubular kidney injury [66]. For female patients, for whom the measurement of a-GalA is not a reliable diagnostic marker, this is especially beneficial. Uromodulin was also tested in ERT patients, and a certain degree of “normalization” was noted, proving the efficacy of the medication [66].

In the French Fabry cohort (FFABRY) study, 40 proteins that are related to inflammatory and angiogenesis processes were measured in plasma samples [69]. This analysis found four proteins with highly significant differential expression levels between the studied groups and phenotype- and sex-related subgroups. This proteomic signature consists of one cytokine and three proteins related to angiogenesis proteins (interleukin 7 (IL-7), vascular endothelial growth factor A (VEGFA), vascular endothelial growth factor C (VEGFC), and fibroblast growth factor 2 (FGF2)). Importantly, female patients had markedly higher concentrations while males had values within the same range. FGF2 is expressed in many tissues and controls cell differentiation and tissue growth during embryonic stages and cellular life (brain, muscles, bones). Strong angiogenic effects, cell growth, and tissue healing are just a few of the biological impacts that FGF2 has when it interacts to the fibroblast growth factor receptor (FGFR) proteins [70]. Numerous disorders, including cancer and cardiovascular conditions, disturb the equilibrium of FGF2. VEGFR2, VEGF, FGF2, phosphorylated p38 mitogen activated protein kinase (p-p38) MAPK, and transforming growth factor beta 1 (TGF-1) expression were all shown to be upregulated in the Fabry mouse kidney, according to Lee et al. Furthermore, these proteins were overexpressed in cultivated endothelium cells after Gb3 therapy [71]. These findings are consistent with the findings from the FFABRY study where the plasma content of FGF2 in Fabry patients was higher than in the controls. FGF2 overexpression also encourages the development and growth of tumors [69]. Based on these studies, blocking of the FGF2 or FGF2/FGFR signaling pathways may slow the growth of tumors [72]. Future research on tumors developed in Fabry patients may assist in clarifying the possible role of FGF2 dysregulation. A novel therapeutic strategy for the treatment of Fabry disease may involve reducing FGF2 plasma levels in Fabry patients. Studies that establish the relationship between FGF2 concentration and symptomatology could also support the potential use of FGF2/FGFR inhibitors in combination with certain treatments [69]. The rise in IL-7 levels seen in the FFABRY study may have a role in the inflammation and impaired autophagy observed in Fabry disease [73,74].

Schiffmann et al. conducted a study in children with Fabry disease where they assessed the effect of ERT during six months on global protein changes by using O-methylisourea-based differential isotope labeling with tandem mass spectrometry (MS/MS). Five proteins following ERT were shown to be: Ig-α-2 C chain, α2-HS glycoprotein, transferrin, α-2-antiplasmin, and vitamin D-binding protein [75]. An increase in circulating VEGF was related to decreased α-2-antiplasmin [75]. Although the cause of the Ig drop is unclear, an immunological reaction is not likely to be the cause. It is intriguing that ERT caused levels of this protein to drop, possibly having an anti-inflammatory impact. Furthermore, the increased levels of vitamin D-binding protein have been linked to various physiological problems, such as endothelial shear stress. Given the considerable vasculopathic component of Fabry disease, endothelial stress is at least partially reduced by ERT with agalsidase alfa [76]. The proliferative properties of the vitamin D-binding protein on vascular smooth muscle cells could contribute to the elevated arterial intimal-medial thickness ratio and myocardial hypertrophy seen in Fabry patients, as well as the growth-promoting effects of plasma from untreated Fabry patients on cultured vascular smooth muscle cells and myocytes. [75]. It is possible to hypothesize that the drop in tissue free iron levels in Fabry disease patients is a result of aberrant cellular turnover or a degree of chronic inflammation. The quantity of free iron available to catalyze the Fenton reaction’s production of reactive oxygen free radicals would typically be reduced by more chelation of free iron caused by higher transferrin levels [75]. Another possible explanation is that Fabry disease may have defective glycosphingolipid recycling, which impairs transferrin recycling via the rab4 pathway. Niemann–Pick A and C disease have been associated with a similar defect [75]. An established plasmin inhibitor from the serpin family is α-2-antiplasmin (serine protease inhibitors) [77]. It is the main inhibitory molecule that regulates fibrinolysis caused by plasmin. Pathognomonic of an overconsumption of these substances is the decrease of plasminogen and α-2-antiplasmin together, which is positively correlated [78]. These anomalies were also observed in individuals who had never had ERT, indicating that they are a feature of the Fabry disease phenotype. The absence of liver dysfunction in Fabry disease and the absence of a correlation between serum albumin levels and α-2-antiplasmin rule out the possibility of a synthetic aberration of fibrinolytic proteins.

Studies that investigated the importance of proteomics in Fabry disease are summarized in Table 2.

## 4. Conclusions

In molecular biology, it is a great challenge to figure out how differences in the genotype lead to a particular phenotype. Proteins are the ultimate expression of genes and have a direct impact on the cell. Proteomics can reveal the problems of how proteins are assembled, kept stable, degraded, and signaled. All of these items can be very important in disease states.

In this review, we tried to show that proteomic profiling in ADPKD and Fabry patients can be used to make diagnostic and prognostic models for these diseases, which could improve the accuracy of the diagnosis, help us learn more about the molecular pathways that cause the disease, monitor patients with rapidly progressive disease, and make it possible for each patient to receive personalized treatment. Developing a urinary exosomal protein expression atlas for ADPKD will facilitate the identification of patients in need of treatment and those most likely to benefit from long-term pharmacotherapy. Hopefully, the combination of newly found and well-known biomarkers of FD, such as proteinuria, GFR decline, and Gb3 levels, could improve the workup, predict how the disease will progress, and estimate the efficacy of the therapy.

Whereas the relevance of proteomics for molecular diagnostics has been demonstrated, additional research is necessary to improve performance and reproducibility between laboratories. However, concerns about the pre-analytical variables, analytical variation, and biological sample variance must be addressed before proteomics tools can be integrated into routine clinical laboratory practice. The digitization of medical care and the use of big data are progressing. There will be changes in how we work, not only in image-based diagnostic areas, such as radiology and pathology, but also in clinical areas. Electronic medical records, omics approaches, genetic maps, and new imaging techniques can provide rich and multidimensional data about inherited diseases.

## Figures and Tables

**Table 1:** Summary of the studies on proteomic analysis, urinary extracellular vesicles (uEVs), and urinary exosomes in autosomal dominant polycystic kidney disease (ADPKD).

Study	Method	Analysis	Results	Conclusion
Kistler et al. [40]	Mass spectrometry-based proteomics	Urinary proteomic biomarkers	Identified over 200 peptides associated with ADPKD; alteration of urinary collagen fragments; upregulation of fibrinogen alpha chain and of keratin; downregulation of c-terminal fragments of uromodulin; increased osteopontin fragments in the urine.	Urinary proteomic biomarkers can improve ADPKD diagnosis and risk stratification for better patient outcomes.
Pejchinovski et al. [47]	Mass spectrometry-based proteomics	Urine peptidome analysis	Identified 20 urinary peptidome biomarkers for predicting end-stage renal disease and ADPKD progression. The biomarker score was equivalent to that of the htTKV. Identified proteolytic pathways involved in ADPKD progression, which could serve as potential targets for therapeutic intervention.	Non-invasive diagnostic tool using urinary biomarkers can predict ADPKD progression and identify targets for therapy.
Rauniyar et al. [49]	Tandem mass tag-based proteomics	Quantification of protein expression	Identified potential urinary protein biomarkers of the cyst growth rate in ADPKD.	Urinary biomarkers could serve as non-invasive tools for ADPKD diagnosis and monitoring.
Salih et al. [54]	Mass spectrometry-based proteomics	Proteomic analysis of urinary extracellular vesicles (uEVs)	Identified potential biomarkers (plakins and complement proteins) and therapeutic targets for ADPKD.	The study provides insight into ADPKD progression and identifies potential targets for therapeutic intervention.
Pocsfalvi G. [53]	Mass spectrometry-based proteomics	EVs isolated from pooled urine samples	Identified 83 differentially expressed extracellular vesicle (EV) proteins involved in signal transduction pathways of primary cilia, Ca(2+)-activated signaling, cell-cycle regulation, and cell differentiation. The reduced levels of AQP-2 and increased levels of APO-A1 indicate impaired renal concentrating capability and may correlate with the decline in eGFR.	Quantitative proteomics of urinary EVs can be a useful tool in studying ADPKD.
Hogan et al. [50]	Electron microscopy and immunoblotting	Characterization of exosome-like vesicles	Identified 552 proteins implicated in signaling; confirmed the cleavage of polycystin-1 and fibrocystin.	Isolation from urine could be a non-invasive method for the diagnosis and monitoring of the disease. The study of PKD-ELVs and their relationship with primary cilia adds a novel aspect to our understanding of polycystic kidney.
Hogan et al. [51]	Mass spectrometry-based proteomics of urinary exosomes	Identification of biomarkers for PKD1 using urinary exosomes	Identified potential biomarkers for ADPKD using urinary exosomes; low PC1/TMEM2 ratios from the start of ADPKD; this ratio may have an inverse relationship with the htTKV.	Urinary exosomal biomarkers may have a clinical utility in the management of ADPKD.

ADPKD-autosomal dominant polycystic kidney disease-ADPKD; PC1/TMEM2 ratios-polycystin-1(PC1)/transmembrane protein 2 (TMEM2); PKD-ELVs-polycystic kidney disease-exosome-like vesicles; AQP-2-aquaporin-2; APO-A1-apolipo protein A1; htTKV-height-adjusted total kidney volume.

**Table 2:** Proteomic studies in Fabry disease.

Authors	Sample	Methods	Number of Patients	Results	Conclusion
Blood derived proteins					
Heo et al. [63]	Blood (before and after ERT)	2D electrophoresis, MALDI-TOF MS, MS/MS.	Eight patients with classical FD.	Pre-ERT significantly increased: ACTB, iC3b, C4B. Following longer-term ERT, iC3b levels gradually decreased and were comparable with Gb3 levels.	C3-mediated complement activation is changed in FD. ERT could promote its stabilisation.
Cigna et al. [67]	Blood (PBMC from FD patients)	2D electrophoresis, MALDI-TOF MS.	Eight FD patients (30–59 years, 6 males and 2 females; 2 patients on ERT) and six healthy controls.	Downregulated proteins: Rho GDP-dissociation inhibitor 2, Rho GDP-dissociation inhibitor 1, calnexin, chloride intracellular channel protein 1. Upregulated proteins: 14-3-3 protein theta, 14-3-3 protein zeta/delta, γ-enolase, galectin-1.	Patients with FD display changes in the PMBC proteome compared to healthy subjects.
Moore et al. [75]	Blood (serum before and after 6 months of ERT)	*O*-methylisourea-based differential isotope labeling with tandem MS (MS/MS)	Thirteen children (6.5–17 years)	Decrease after ERT: α2-HS glycoprotein, transferrin, vitamin D-binding protein, α-2-antiplasmin, Ig-α-2C chain. Decreased α-2-antiplasmin was associated with an increase in circulating VEGF.	Present abnormalities of angiogenesis factors and fibrinolysis.
Hollander et al. [79]	Blood	LC-MS/MS, iTRAQ	Thirty-two FD patients; 14 healthy controls.	Proteins sensitive and specific for male patients: afamin, peroxiredoxin 2, haemoglobin α-2, 22 kDa protein, α1 antichymotrypsin, β-Ala His dipeptidase, apolipoprotein E, isoform 1 of sex hormone-binding globulin. Protein biomarker panel in female patients: paraoxonase 1, kallistatin, isoform 1 of gelsolin, epithelium-derived factor, haemoglobin subunit apha/haemoglobin alpha2, apolipoprotein E, alpha-cardiac muscle 1 actin, peroxiredoxin 2, pigment protein *Z*-dependent protease inhibitor.	Gender-specific plasma protein biomarker panels were identified.
Urine derived proteins					
Matafora et al. [66]	Urine	LC-MS/MS	Eleven FD patients non-ERT treated and twelve ERT-treated patients; twelve healthy controls.	Upregulated proteins: prostaglandin H2 d-isomerase, uromodulin, prosaposin.	The urinary proteome of FD patients is different from healthy controls; upregulated proteins are decreased after ERT.
Doykov et al. [80]	Urine	LC-MS/MS	Sixty-six patients (27 males, 39 females)	Urinary proteins elevated in the early stage/asymptomatic patients: uromodulin, glycogen phosphorylase brain form, albumin, endothelial protein receptor C, α1-antitrypsin, intracellular adhesion molecule 1. Proteins elevated only in patients with renal involment: FGF23, podocalyxin, AMBP, cubulin. Increased in all symptomatic patients: nephrin, prosaposin.	Glycogen phosphorylase brain form was the only protein elevated from the early-stage and continued to increase with progressive multiorgan involvement. Protein biomarkers might be used for the monitoring of therapy or disease progression.
Kistler et al. [81]	Urine	CE-MS; Micro-TOF MS	Thirty-five FD female patients (non-treated); eighty-nine healthy controls.	Sixty-four identified diagnostic biomarkers (88.2% sensitivity, 97.8% specificity)	Urinary biomarker model performing well in diagnosis of FD in female patients and in monitoring the response to ERT.
Manwaring et al. [82]	Urine	LC-MS/MS	Ten pediatric FD male patients; 6–16 years.	Prosaposin and GM_2_AP were elevated in FD patients and reduced after 12 months of ERT.	Protein biomarkers could be used for monitoring the response to ERT.
Vojtová et al. [83]	Urine (comparison between FD patients and healthy controls)	2D electrophoresis images of urine samples, MALDI-TOF MS	Twenty FD patients (18–69 years; 11 males, 9 females), 13 patients were on ERT, Ten control subjects (27–42 years; 5 males, 5 females).	Abundant proteins in FD patients were: alpha-1-antitrypsin, alpha-1-microglobulin, Ig kappa chain V–III, complement-c1q tumor necrosis factor-related protein, prostaglandin H2 d-isomerase.	No significant qualitative differences between treated and untreated FD patients. Molecular size of H2 d-isomerase was modified.
Proteins derived from cell model					
Neto et al. [84]	Cell model (human podocytes)	Two-dimensional differential gel electrophoresis (2D-DIGE), MALDI-TOF MS.	Not applicable.	Downregulated proteins: HSPD1, UCHL1, enolase 1. Overexpressed proteins: vimentin, TGF-β, VEGF-2, PI3K 110a, PI3K 110b.	FD podocytes express a profibrotic proliferative pattern.
Slaats et al. [85]	Urine-derived cells	nLC-MS/MS	Seven patients (5 males and 2 females; 26–68 years)	Increased proteins: FBN1, SPG20, GOCC, INA, TFRC, PTPN23, TOR4A.	Urine-derived cells from FD patients could be used as diagnostic tools, ERT monitoring, and testing therapeutic interventions.
Renal biopsy					
L’Imperio et al. [86]	Renal biopsy	MALDI-TOF MS, MALDI MS/MS, MALDI-MSI.	Fourteen FD patients (6 males, 8 females, 19–66 years)	Differences in protein expression between female and male FD patients, as well as between classic and atypical variants.	MALDI-MSI allows for phenotypic distinction in FD and possibility of genetic classification.

Legend: MALDI-TOF MS-matrix-assisted laser desorption/ionization time-of-flight mass spectrometry; MALDI-MSI-matrix-assisted laser desorption/ionization mass spectrometry imaging; nLC-nano-liquid-chromatography; LC-MS/MS-liquid chromatography and tandem mass spectrometry; CE-MS-capillary electrophoresis-mass spectrometry; iTRAQ-isobaric tags for relative and absolute quantification; ERT-enzyme replacement therapy; iC3b-inactivated complement C3b; ACTB-β-actin; Gb3-globotriaosylceramide; VEGF-vascular endothelial growth factor; PBMC-peripheral blood mononuclear cells; GM_2_AP-GM2 activator protein; FGF23-fibroblast growth factor 23; AMBP-alpha-1-microglobulin/bikunin precursor; GOCC-lysosomal lumen proteins; FBN1-fibrillin; INA-alpha-internexin; SPG20-spartin; PTPN23-protein tyrosine phosphatase non-receptor type 23; TFGR-transferrin receptor; TOR4A-torsin family 4 member A.HSPD1-heat shock protein family D member 1; UCHL1-ubiquitin c-terminal hydrolase L1; TGF-β-transforming growth factor beta; PI3K-phosphoinositide 3-kinase.
